# Bacterial Meningitis in Brazil: Baseline Epidemiologic Assessment of the Decade Prior to the Introduction of Pneumococcal and Meningococcal Vaccines

**DOI:** 10.1371/journal.pone.0064524

**Published:** 2013-06-18

**Authors:** Luciano Cesar Pontes Azevedo, Cristiana M. Toscano, Ana Luiza Bierrenbach

**Affiliations:** 1 Research and Education Institute (IEP), Hospital Sírio-Libanês, São Paulo, Brazil; 2 Emergency Medicine Department, University of São Paulo, Brazil; 3 Department of Collective Health, Federal University of Goiás, Goiânia, Brazil; 4 Sanas Epidemiology and Research, São Paulo, Brazil; Centers for Disease Control & Prevention, United States of America

## Abstract

**Background:**

Bacterial meningitis is associated with significant burden in Brazil. In 2010, both 10-valent pneumococcal conjugate vaccine and meningococcal capsular group C conjugate vaccine were introduced into the routine vaccination schedule. *Haemophilus influenzae* type b vaccine was previously introduced in 1999. This study presents trends in demographics, microbiological characteristics and seasonality patterns of bacterial meningitis cases in Brazil from 2000 to 2010.

**Methods and Findings:**

All meningitis cases confirmed by clinical and/or laboratory criteria notified to the national information system for notifiable diseases between 2000 and 2010 were analyzed. Proportions of bacterial meningitis cases by demographic characteristics, criteria used for confirmation and etiology were calculated. We estimated disease rates per 100,000 population and trends for the study period, with emphasis on *H. influenzae*, *N. meningitidis* and *S. pneumoniae* cases. In the decade, 341,805 cases of meningitis were notified in Brazil. Of the 251,853 cases with defined etiology, 110,264 (43.8%) were due to bacterial meningitis (excluding tuberculosis). Of these, 34,997 (31.7%) were due to meningococcal disease. The incidence of bacterial meningitis significantly decreased from 3.1/100,000 population in 2000–2002 to 2.14/100,000 in 2009–2010 (p<0.01). Among cases of meningococcal disease, the proportion of those associated with group C increased from 41% in 2007 to 61.7% in 2010, while the proportion of group B disease progressively declined. Throughout the study period, an increased number of cases occurred during winter.

**Conclusions:**

Despite the reduction in bacterial meningitis incidence during the last decade, it remains a significant healthcare issue in Brazil. Meningococcal disease is responsible for the majority of the cases with group C the most common capsular type. Our study demonstrates the appropriateness of introduction of meningococcal vaccination in Brazil. Furthermore, this study provides a baseline for future evaluation of the impact of the vaccines introduction in Brazil and changes in disease epidemiology.

## Introduction

In Brazil, surveillance of meningitis is based on mandatory notification of all suspected cases occurring in the public or private healthcare systems, including healthcare units and diagnostic laboratories. Cases are notified to the national information system for notifiable diseases (“Sistema de Informação de Agravos de Notificação” - SINAN), a national case-based information system managed by the Brazilian Ministry of Health. SINAN was implemented countrywide in the early 90s and has gone through several improvements, the last one in 2007 when it shifted to an internet-based data transfer system along with other minor changes [1].

Meningitis surveillance is critical for the timely detection of epidemics and for determining the local disease burden, so that proper prevention and control strategies can be implemented. The main pathogens causing bacterial meningitis are *H. influenzae*, *N. meningitidis* and *S. pneumoniae*, for which conjugate vaccines are now available globally. In this regard, *H. influenzae* type b (Hib) vaccine was introduced into the routine vaccination schedule in Brazil in 1999, and more recently, both 10-valent pneumococcal (PCV10) and meningococcal capsular group C (MenC-V) conjugate vaccines were introduced in 2010. This study describes the epidemiology of bacterial meningitis across all age groups in Brazil in the decade prior to the introduction of these new vaccines, with a particular focus on meningococcal disease. The epidemiological scenario that motivated the incorporation of these new vaccines into the routine schedule, taking into consideration both the optimal age for vaccination and the potential coverage against prevalent serogroups is discussed, so that future studies can benefit from our baseline assessment in order to measure the impact of these interventions.

## Methods

### Ethics Statement

Ethical approval for this study was obtained from the Ethical Committee of Hospital Sírio-Libanês, São Paulo, Brazil (Comissão de Ética em Pesquisa - CAAE: 01063212.1.0000.5461 - 01/03/2012). Nominal information was used only for record-linkage purposes in order to clean the database of duplications. Since this is an observational analysis of a national secondary database, subject's informed consent was waived from the Institutional Ethics Committee.

### Methods

All meningitis and meningococcemia cases confirmed by clinical and/or laboratory criteria reported to SINAN between 2000 and 2010 were included in the study. Case-based variables available for analysis include demographic, clinical and microbiological information.

Laboratory and/or clinical/epidemiologic criteria were considered for definition of meningitis cases, following the recommendations provided by the Brazilian Health Ministry guidelines [2]. Accordingly, a suspected case was defined as any children and adults of any age, for whom the attending physician suspected of meningitis. Clinical signs and symptoms included fever, headache, vomit, neck stiffness, signs of meningeal irritation, seizures, and/or rash. In children younger than 1 year of age, unspecific symptoms were considered. Laboratory confirmation included the following specific methods: culture or antigen detection in the cerebrospinal fluid (CSF), including latex or contraimmuno-electrophoresis tests. Although other laboratory confirmation tests could be used, including Enzyme-linked immunosorbent assay (ELISA), polymerase chain reaction (PCR) and immunofluorescence, these are mostly used in academic institutions and are not routinely available. Suspected cases that had contact to a laboratory-confirmed case were confirmed through clinical/epidemiological criteria. Meningococcemia suspected cases could be confirmed by CSF inspection showing gram-negative diplococcic and petequiae.

As SINAN is a nationwide system, which sensitivity and representativeness for meningitis has been demonstrated to be high [3], [4], in this analysis we refer to notification rates as incidence rates.

Annual bacterial meningitis incidence rates per 100,000 population, by age group, gender, pathogen, and serogroup were calculated. Population estimates were provided by the Brazilian Institute of Geography and Statistics (“Instituto Brasileiro de Geografia e Estatística” – IBGE). Age groups considered were those recommended by World Health Organization (WHO) for assessing meningitis surveillance data: 0–23 months, 2–4 years, 5–14 years, 15–29 years, 30–45 years and 45 years and more [5]. Monthly population data by age groups were estimated by linearly interpolating the mid-year estimates for the months in between. All analyses were done using Stata 12 (Stata Corporation, Texas, USA). Temporal trends were assessed using the non-parametrical test for trend across ordered groups (nptrend command in Stata), with a P value considered statistically significant when below 0.05.

SINAN upgraded its operational system in 2006/2007. For some months the two systems were running in parallel, so that cases could have been notified to both systems, generating duplicate records. Duplicate records may also have been generated when cases were transferred within (e.g. one notification from the emergency room and another from the intensive care unit) or in between health care units. In order to identify and exclude duplicated cases from our analysis, a three-step process was applied. First, an in-house deterministic record linkage algorithm, similar to the one validated by Pacheco et al [6] was used to find records that belonged to the same patient. Next, we considered that consecutive records of the same patient with an interval of up to seven days between the dates of entry belonged to the same episode of disease. We then discarded repeated records of the same episode of disease. For cases that had different disease etiology information recorded in the duplicated records, we applied the following exclusion priority rules: missing values>unspecified> other etiology (parasitic and fungal diseases)>viral>tuberculosis>*H. influenzae*>pneumococcal>meningococcemia>meningococcal meningitis>meningococcal meningitis with meningococcemia. Records with higher priority for exclusion were removed from the analysis.

Guidelines for completion of some variables were modified in 2006/2007. Most notably, the main criteria used for case confirmation, i.e. whether it was based on laboratory or clinical/clinical-epidemiological findings, became more stringent. As a consequence, a decrease in the number of cases classified as “unspecified bacteria” (cases marked as having a bacterial disease due to “other” bacteria for which no etiology was specified in the corresponding field) and an increase in the number of cases classified as “unspecified etiology” was observed. We thus present data on the criteria used for diagnosis by pathogen only for 2007–2010.

As information on *N. meningitidis* capsular groups only started to be recorded after the system upgrade in 2007, we present data on *N. meningitidis* capsular groups by notification year and age groups only from 2007 onwards. Information on *S. pneumoniae* serotypes is not available in SINAN.

## Results

Over the 11-year period, 341,805 cases of meningitis and meningococcemia were notified to SINAN, and of these 327,449 (95.8%) had CSF collected. Of these, 251,853 (73.7%) cases had a defined etiology, and 89,952 (26.3%) were pathogen unspecified. Of the 251,853 cases with a defined etiology, 110,264 (43.8%) were classified as bacterial meningitis cases (excluding tuberculosis), 125,881 (49.9%) as aseptic, 11,546 (4.6%) as fungal/parasitic, and 4,162 (1.7%) as tuberculosis.

Of the 110,264 bacterial meningitis cases, 34,997 (31.7%) were due to *N. meningitidis*, 13,209 (12%) were due to *S. pneumoniae*, 2,152 (2%) to *H. influenzae* type b, 4,749 (4.3%) to other bacteria, and 55,157 (50%) had no bacteria specified.

From the 34,997 meningitis cases due to N. meningitidis, 9,999 (28.6%) were classified as meningococcemia, 13,720 (39.2%) as meningococcal meningitis, 11,278 (32.2%) as meningococcemia combined with meningococcal meningitis. We will refer to these three combined categories as cases of meningococcal disease.

Among the 4,749 cases with other bacteria specified, the most common ones were *Staphylococcus* (including *S. aureus*, *S. epidermidis*, and *Staphylococcus sp.*) (988 cases, 20.8%), *Streptococcus* (including *S. pyogenes*, *S. agalactie*, and *Streptococcus sp.*) (656 cases, 13.8%), and *Acinetobacter* (including *A. baumannii*, and *Acinetobacter sp.*) (295 cases, 6.2%). Only 47 (1%) cases were due to *Listeria monocytogenes*, of which only 3 were in the neonatal period.

**Figure 1:**
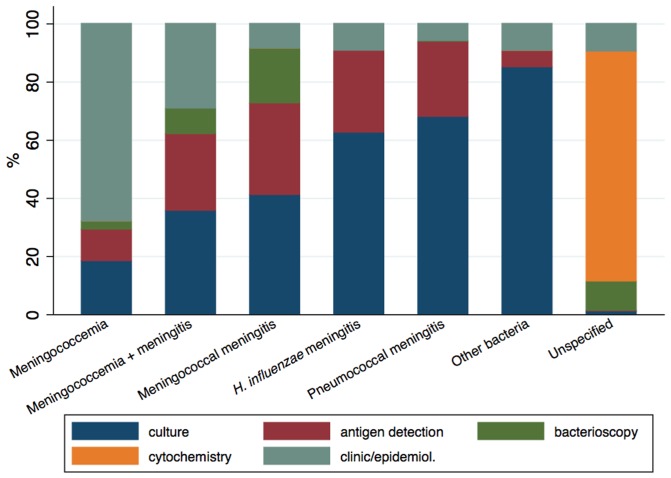
Proportion of bacterial meningitis cases notified to SINAN in Brazil from 2007 to 2010 by criteria used for case confirmation, according to pathogen. Antigen detection methods include latex agglutination, counterimmunoelectrophoresis (CIE) and polymerase-chain reaction (PCR).

Of the 110,264 bacterial meningitis cases, 30,237 occurred from 2007 onwards. Of these, 11,097 (36.7%) were confirmed by culture, 7,529 (24.9%) by cytochemical analysis, 5,291 (17.5%) by clinical/epidemiological criteria, 4,142 (13.7%) by antigen detection methods and 2,178 (7.2%) by bacterioscopy. These percentages add up to 100% as in SINAN only one single methodology representing the main method used for case confirmation is informed for each case. During this period, the proportion of cases confirmed by culture, bacterioscopy and by clinical symptoms remained relatively stable, whereas cases confirmed by antigen detection methods increased from 10.2% in 2007 to 17.9% in 2010 and cases confirmed by cytochemical analysis decreased from 29% in 2007 to 22% in 2010. Nonetheless, the main method used for diagnosis varied according to the pathogen (Figure 1). While the diagnosis of meningococcemia was mostly based on clinical/epidemiological criteria, the diagnosis of meningitis due to unspecified bacteria was, as expected, mostly dependent on CSF cytochemical results. Culture contributed to the diagnosis of less than 40% of meningococcemia/meningitis due to *N. meningitidis* and to over 60% for the other specified bacteria.

**Figure 2:**
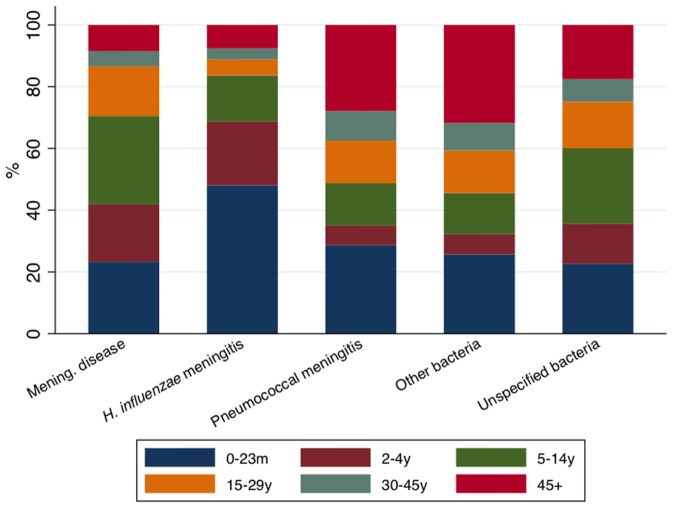
Proportion of bacterial meningitis cases notified to SINAN in Brazil from 2000 to 2010 by age group, according to the pathogen.

Figure 2 shows that the age group of the bacterial meningitis cases varied according to the pathogen. Almost half of the cases due to *H. influenzae* were under two years of age, while for the other pathogens this proportion varied in between 20% and 30%. The proportion of cases in adults aged 30 years and over was higher for cases due to *S. pneumoniae* than for those due to *N. meningitidis* and *H. influenzae*, and similar to the proportion for all the other specified bacteria combined (around 40%).

Incidence rates of meningitis cases caused by *N. meningitidis*, *S. pneumoniae* and *H. influenzae*, stratified by age group, gender, and pathogen are presented in Table 1. Cases of meningococcal disease still predominate as compared to the other bacteria. A 30% decline in bacterial meningitis incidence overall is observed from 2000–2002 (3.09 cases/100,000 population) to 2009–2010 (2.15 cases/100,000 population) (p<0.01). Considering the various bacterial etiologies, the most significant proportional decline was observed for *H. influenzae* cases (72%), which is a result of Hib vaccine introduction in 1999. Incidence rates were significantly higher in males for all the three pathogens analyzed. Disease incidence was higher in children below 5 years of age for all the three pathogens analyzed, with a lower incidence in young adults and again an incidence peak in the age group of 45 years of age and older, mainly represented by *S. pneumoniae* cases.

**Table 1:** Incidence rates of meningitis due to *N. meningitidis*, *S. pneumoniae* e *H. influenzae* per 100,000 population in Brazil, 2000 to 2010, stratified by age group, gender, and pathogen.

	Rates/100,000 population				
Characteristic	2000–2002	2003–2005	2006–2008	2009–2010	p-value for trend*
**Pathogen**					
All three pathogens	3.10	2.63	2.20	2.14	0.003
*Neisseria meningitidis*	2.21	1.81	1.48	1.51	0.004
Meningococcemia	0.57	0.52	0.44	0.49	0.010
Meningococcal meningitis	0.86	0.72	0.59	0.57	0.004
Meningococcemia+meningitis	0.78	0.58	0.45	0.45	0.003
*Haemophylus influenzae*	0.21	0.08	0.07	0.06	0.005
*Streptococcus pneumoniae*	0.67	0.74	0.65	0.58	0.069
**Age group**					
*Neisseria meningitidis*					
0–23 m	9.81	7.68	6.29	7.05	0.007
2–4 y	12.55	12.45	10.47	8.54	0.078
5–14 y	3.34	2.85	2.35	2.35	0.004
15–29 y	1.14	0.98	0.88	1.06	0.053
30–45 y	0.39	0.39	0.36	0.44	0.751
45+	0.6	0.57	0.58	0.62	0.583
*Haemophylus influenzae*					
0–23 m	1.92	0.65	0.55	0.57	0.009
2–4 y	1.49	0.4	0.54	0.35	0.043
5–14 y	0.15	0.08	0.06	0.06	0.009
15–29 y	0.03	0.01	0.02	0.02	0.544
30–45 y	0.02	0.02	0.01	0.02	0.204
45+	0.04	0.04	0.03	0.03	0.010
*Streptococcus pneumoniae*					
0–23 m	4.09	3.74	3.45	2.98	0.003
2–4 y	1.16	1.64	1.5	1.39	0.078
5–14 y	0.48	0.56	0.49	0.45	0.686
15–29 y	0.32	0.37	0.34	0.29	0.341
30–45 y	0.28	0.33	0.3	0.24	0.583
45+	0.7	0.86	0.73	0.72	0.751
**Gender**					
*Neisseria meningitidis*					
Male	2.45	2.05	1.68	1.74	0.004
Female	1.96	1.58	1.3	1.28	0.004
*Haemophylus influenzae*					
Male	0.24	0.1	0.07	0.08	0.009
Female	0.19	0.06	0.07	0.04	0.007
*Streptococcus pneumoniae*					
Male	0.84	0.88	0.81	0.7	0.057
Female	0.51	0.6	0.49	0.46	0.175

* Notification years (and not notification sub-periods shown on this table) were used as the group variable of the non-parametric tests.

**Figure 3:**
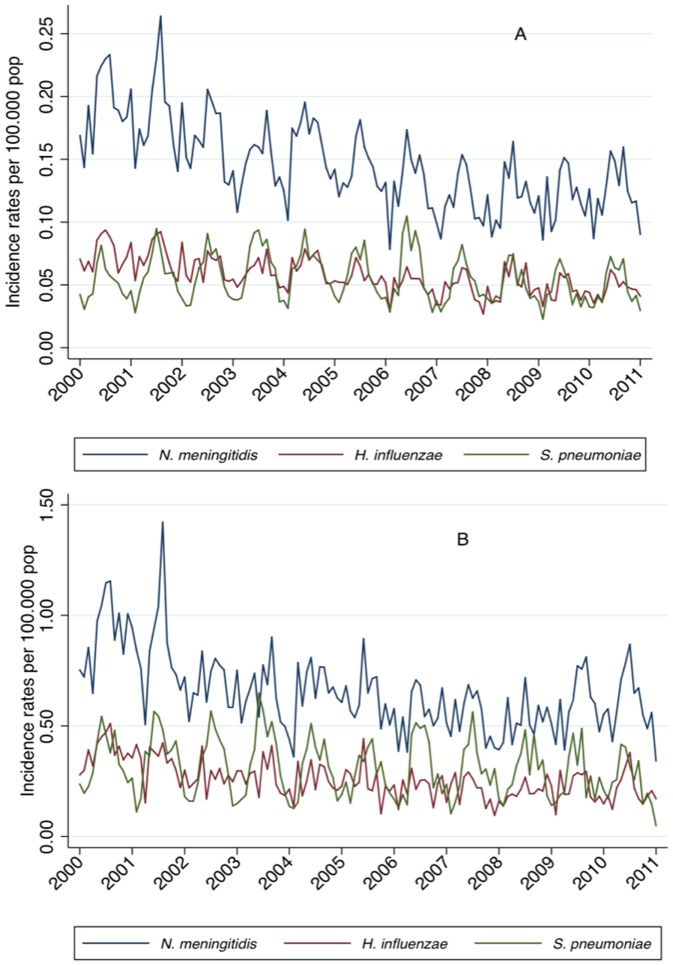
Monthly incidence rates of meningitis due to *N. meningitidis*, *S. pneumoniae* and *H. influenzae* per 100,000 population, by age-group, in Brazil, 2000 to 2010. Panel A: All age groups combined, Panel B: Children aged 0 to 23 months.

The incidence of meningitis cases due to *N. meningitidis*, *S. pneumoniae* and *H. influenzae* decreased over time. A marked seasonal pattern was observed with increased incidence during autumn-winter periods (from May to October) and decreasing in the summer. Incidence rates for all pathogens were markedly higher in children younger than 2 years of age. In this age group, we can observe that the incidence of *N. meningitidis*, more evidently, and of *H. influenzae* had a decreasing trend up to 2006, after which they tended to stabilize. In this age group we also observed that *S. pneumoniae* meningitis, in particular, decreased after 2010 (Figure 3).

**Figure 4:**
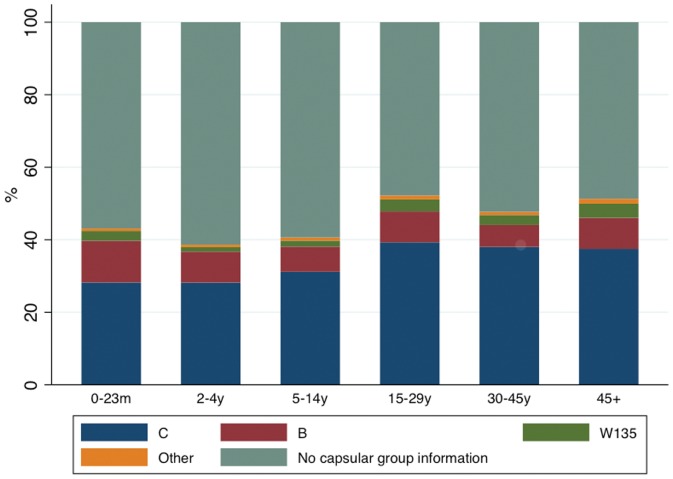
Distribution of meningococcal disease cases by capsular subgroup (n = 11,037) in Brazil, 2007 to 2010.

Out of the 11,037 meningococcal disease cases notified from 2007 onwards, 6,137 (55.6%) had no capsular group information recorded, 3,588 (32.5%) were due to group C (MenC), 949 (8.6%) were due to group B (MenB), 264 (2.4%) to group W135 (MenW) and only 99 (0.9%) were due to the other groups (A, D, X, Y, Z and 29E). If we consider only the 5,528 cases (out of the 11,037) for which the criteria used for diagnosis was recorded as culture, latex agglutination or PCR, the proportion of those with no information about capsular group is reduced to 27.5%, and more than half (52.6%) were due to MenC. This proportion progressively increased from 41% in 2007 to 61.7% in 2010, while the proportion of MenB progressively decreased (Table 2). The capsular group distribution did not vary by age (Figure 4).

**Table 2:** Distribution of laboratory confirmed *N. meningitidis* cases by capsular group in Brazil, 2007 to 2010.

Year	Capsular group n (%)									Total
	A	B	C	X	Y	Z	W 135	29 E	No information	
2007	1 (0.1)	232 (19.4)	491 (41)	0 (0)	8 (0.7)	0 (0)	32 (2.7)	0 (0)	433 (36.1)	1,197
2008	5 (0.4)	217 (16.4)	665 (50.2)	1 (0.1)	9 (0.7)	1 (0.1)	66 (5)	1 (0.1)	356 (27.0)	1,321
2009	8 (0.6)	183 (13.5)	736 (54.1)	0 (0)	15 (1.1)	0 (0)	53 (3.9)	0 (0)	365 (26.8)	1,360
2010	4 (0.2)	178 (10.8)	1,018 (61.7)	0 (0)	11 (0.7)	0 (0)	70 (4.2)	3 (0.2)	366 (22.2)	1,650
Total	18 (0.3)	810 (14.7)	2,910 (52.6)	1 (0)	43 (0.8)	1 (0)	221 (4)	4 (0.1)	1,520 (27.5)	5,528

Laboratory criteria included culture, latex agglutination or polymerase chain reaction.

## Discussion

Bacterial meningitis is a significant cause of death and disability in children below five years of age in the world [7]–[12]. In most countries *N. meningitidis* is a leading cause of meningitis and fulminant sepsis, but, according to the WHO, no reliable global disease burden estimate is currently available [13]. Invasive meningococcal infections are usually caused by serogroups A, B, C, W135, X, or Y. Their relative prevalence varies with time and geographic location. While serogroup A meningococci cause large epidemics in sub-Saharan Africa, serogroups B and C organisms are responsible for sporadic cases and localized disease outbreaks worldwide [13].

Our study used secondary data to evaluate meningitis epidemiology and disease burden in Brazil. This allowed us to analyze data from a long time series, which is representative of the whole population. Despite a considerable decline in the number of cases in the last decade, the three main pathogens are still responsible for a significant incidence, mainly in children below four years of age and in particular *N. meningitidis*. In addition, we demonstrated that serogroup C is the most important capsular type among *N. meningitidis* cases in Brazil.

Our analysis demonstrates a lower incidence of bacterial meningitis in Brazil when compared with other low or middle-income countries. While in India, a higher incidence of meningococcal disease, mostly due to epidemics caused by MenA [14] has been described, our study demonstrate an increased incidence of MenC and decline in the number of cases of MenB in Brazil. Interestingly, the incidence of meningococcal disease in India seems to be lower than the caused by other agents as *S. pneumoniae* or *H. influenzae* [14]. In Cuba, Pérez et al demonstrated overall incidence rates of bacterial meningitis similar to those described in our study for Brazil. However, most cases in Cuba are due to S. *pneumoniae*, and periodic vaccination campaigns may have been responsible for the lower proportion of cases caused by *N. meningitidis* [15].

When compared to developed countries, the incidence of bacterial meningitis in Brazil is still high. Data from the USA prior to PCV introduction demonstrate a reduction in the number of meningitis cases from 1998 to 2007, with a higher proportion of cases due to *S. pneumoniae*. The age distribution of cases in the USA was similar to the observed in our study, with a reduction of incidence in young adults and another peak of incidence in older persons, mainly due to pneumococcal disease [10]. In a 31-year descriptive study, Stanton et al characterized the cases of meningococcal disease admitted to a tertiary children center in England and identified a significant proportion of cases caused by MenB with the majority of cases occurring below 4 years of age. In this study, the proportion of cases due to MenC increased significantly until the year 2000, when meningococcal vaccination reduced the number of cases to very low levels [16].

Prior to Hib vaccine introduction in Brazil, the epidemiology of bacterial meningitis was characterized by the predominance of meningococcal disease and *H. influenzae* meningitis over cases caused by *S. pneumoniae* [17], [18]. Various Brazilian studies focusing on *N. meningitidis* confirms our findings in term of serogroups most commonly identified. While some authors demonstrate the predominance of MenB cases in the late 90's and early 2000 [19], [20], more recent evaluations indicate that most cases of meningococcal disease in the last decade were caused by MenC [21].

The introduction of Hib vaccination significantly reduced the incidence of invasive disease caused by *H. influenzae* in several high-income and developing countries [22]–[24]. In Brazil, a surveillance study carried out in Salvador, Bahia, demonstrated a considerable reduction in the incidence of Hib meningitis mainly in children below one year of age (from 60.9 to 3.1 cases per 100,000 population) [25]. National data from SINAN demonstrated that, prior to Hib vaccine introduction in 1999, *H. influenzae* was responsible for approximately 1,700 meningitis cases every year, with an average annual incidence in all age groups of 1.1 cases/100,000 population in Brazil [26]. After vaccine introduction, a 90% reduction of cases, incidence and deaths was demonstrated. Our study confirms these findings, with a demonstrated 72% incidence reduction of *H. influenzae* meningitis in the entire country in the decade after introduction of the vaccine, most notably in children below two years of age. The almost complete exclusion of Hib as a cause of invasive disease is associated, however, with the phenomenon of serotype replacement. Ribeiro et al observed in their sample a transient increase in the number of cases caused by *H. influenzae* type a [25]. Other subsequent study demonstrated a significant increase in the number of non-b and nontypeable *H. influenzae* meningitis isolates in the years 2000–2008 in a national reference laboratory [23]. This fact may be due to improvements in the surveillance of this pathogen in the period.

Previous reports demonstrated the considerable impact of introducing both pneumococcal and meningococcal vaccine into the routine schedule on the incidence of meningitis in several countries [10], [27]–[29]. Currently licensed meningococcal conjugate vaccines are either monovalent (A or C) or quadrivalent (A,C,Y,W-135) [13]. MenC-V was first licensed in England in 1999 for children aged 2 months and older and adults. In 2005, the quadrivalent meningococcal conjugate vaccine was licensed for use in adolescents and adults in the USA. Large scale MenC-V vaccination has drastically reduced the incidence of MenC-disease in several countries [13], [16], [27].

Most countries in the world implemented meningococcal outbreak control strategies including vaccination of those at risk or exposed during the outbreak. As part of the national disease prevention and control strategies, Brazil has an effective meningitis control program, which includes timely detection of outbreaks, chemoprophylaxis of cases' contacts and vaccination for outbreak control when appropriate [2], [30]. Since mid-70's, vaccines used for outbreak control in the country have been the polysaccharide vaccine types A and C.

In addition to outbreak control, many countries have introduced meningococcal vaccination to high risk individuals such as travelers to areas in which meningococcal disease is hyperendemic or epidemic, patients with anatomic or functional asplenia, and patients with humoral immunodeficiencies, particularly those with complement deficiency [31], [32]. In Brazil, MenC vaccine is provided since 2003 to the above groups, and also to HIV-infected individuals younger than 13 years, patients with cochlear implant, and patients with storage diseases. Efficient outbreak control strategies and vaccination of high-risk groups might be at least partially responsible for the decreasing trends observed for meningococcal disease rates during the initial period of our study. A decreasing trend in the number of newborns from 2006 onwards might be somewhat responsible for the stabilization of the incidence rates after this year.

World Health Organization recommends the introduction of large-scale meningococcal vaccination programs in countries with high (<10 cases/100,000 population/year) or intermediate (2–10 cases/100,000 population/year) endemic rates of invasive meningococcal disease [13]. Regarding pneumococcal vaccination, a heptavalent pneumococcal conjugate vaccine (PCV7) was licensed in 2000 followed by 10-valent (PCV10) and 13-valent (PCV13) PCVs in 2009 and 2010. WHO also recommended the use of these vaccines, especially in countries with high child mortality [33].

Thus, it is expected that the recent introduction of MenC-V [34] and PCV10 [35] in the Brazilian routine immunization schedule will substantially reduce the incidence of these diseases in the coming years. Despite its very recent introduction, it was demonstrated that PCV10 introduction in 2010 resulted in a significant early impact in reducing pneumonia cases in Brazil [36]. Similar early impact in pneumococcal meningitis cases may be demonstrated in future studies. Likewise, it will be important to evaluate pneumococcal serotype replacement in the country following PCV10 introduction. Current evidence does not indicate that significant replacement takes place after meningococcal conjugate vaccine introduction [37].

There are several limitations to our study, mainly due to the use of secondary data sources. Studies that make use of secondary surveillance data such as SINAN are very much contingent on the quality and methods used to collect and classify the data [38]. Surveillance information are much more incomplete and subject to systematic and random errors when compared with data from observational studies, especially prospective ones. Secondary data usually do not allow significant characterization of the study population and selected important information are not adequately represented [4]. On this account, we did not have etiologic information on a significant percentage of patients and outcome data was not available for 74% of the reported cases. Thus, we did not consider the reported 4.8% case fatality ratio to be representative of the population, even as it is more likely that cases that died had their outcome reported. The lack of availability of quality survival information due to incompleteness of the forms is an important limitation of our analysis.

Is SINAN meningitis data good enough for what it is expected to do? Despite its limitations, secondary surveillance data enabled us to track variations in the incidence of the disease and prevalent serogroups, and highlight any significant trends. Also, the availability of such data can be useful to compare observed and projected values as a strategy to evaluate the impact of interventions like vaccination on disease burden.

## Conclusion

Meningococcus and pneumococcus are still responsible for a significant burden of bacterial meningitis in Brazil. MenC is the most prevalent serogroup among meningococcal disease cases, indicating the appropriateness of MenC vaccine introduction in Brazil. In this regard, this study provides important baseline for future evaluation of the impact of PCV10 and MenC vaccine initiation and changes in disease epidemiology, including etiology, serogroup, and age distribution of cases.

## References

[1] Brasil, Ministério da Saúde (2012) O que é o Sinan. Brasília.

[2] Brasil, Ministério da Saúde (2009) Guia de Vigilancia Epidemiológica. Brasilia.

[3] Escosteguy CC, Medronho R deA, Madruga R, Dias HG, Braga RC, et al (2004) [Epidemiologic surveillance and evaluation of meningitis hospital care]. Rev Saude Publica 38: 657–663. 15499436 10.1590/s0034-89102004000500007

[4] Figueira GCNC, Carvalhanas TRMP, Okai MIG, Yu ALF, Liphaus BL (2012) Avaliação do sistema de vigilância das meningites no município de São Paulo, com ênfase para doença meningocócica. Boletim Epidemiologico Paulista 9: 5–25.

[5] World Health Organization (2003) WHO–recommended standards for surveillance of selected vaccine-preventable diseases. In: Department of Vaccines and Biologicals, editor: World Health Organization.

[6] Pacheco AG, Saraceni V, Tuboi SH, Moulton LH, Chaisson RE, et al (2008) Validation of a hierarchical deterministic record-linkage algorithm using data from 2 different cohorts of human immunodeficiency virus-infected persons and mortality databases in Brazil. Am J Epidemiol 168: 1326–1332. 18849301 10.1093/aje/kwn249 PMC2638543

[7] Brouwer MC, Tunkel AR, van de Beek D (2010) Epidemiology, diagnosis, and antimicrobial treatment of acute bacterial meningitis. Clin Microbiol Rev 23: 467–492. 20610819 10.1128/CMR.00070-09 PMC2901656

[8] Kim KS (2010) Acute bacterial meningitis in infants and children. Lancet Infect Dis 10: 32–42. 20129147 10.1016/S1473-3099(09)70306-8

[9] Liu L, Johnson HL, Cousens S, Perin J, Scott S, et al (2012) Global, regional, and national causes of child mortality: an updated systematic analysis for 2010 with time trends since 2000. Lancet 379: 2151–2161. 22579125 10.1016/S0140-6736(12)60560-1

[10] Thigpen MC, Whitney CG, Messonnier NE, Zell ER, Lynfield R, et al (2011) Bacterial meningitis in the United States, 1998–2007. N Engl J Med 364: 2016–2025. 21612470 10.1056/NEJMoa1005384

[11] Ladhani SN, Lucidarme J, Newbold LS, Gray SJ, Carr AD, et al (2012) Invasive meningococcal capsular group Y disease, England and Wales, 2007–2009. Emerg Infect Dis 18: 63–70. 22261040 10.3201/eid1801.110901 PMC3310110

[12] Traore Y, Tameklo TA, Njanpop-Lafourcade BM, Lourd M, Yaro S, et al (2009) Incidence, seasonality, age distribution, and mortality of pneumococcal meningitis in Burkina Faso and Togo. Clin Infect Dis 48 Suppl 2: S181–189. 19191614 10.1086/596498

[13] World Health Organization (2011) Meningococcal vaccines: WHO position paper. Weekly epidemiological record 86: 521–539.22128384

[14] Sinclair D, Preziosi MP, Jacob John T, Greenwood B (2010) The epidemiology of meningococcal disease in India. Trop Med Int Health 15: 1421–1435. 21054695 10.1111/j.1365-3156.2010.02660.x

[15] Perez AE, Dickinson FO, Rodriguez M (2010) Community acquired bacterial meningitis in Cuba: a follow up of a decade. BMC Infect Dis 10: 130. 20500858 10.1186/1471-2334-10-130 PMC2891755

[16] Stanton MC, Taylor-Robinson D, Harris D, Paize F, Makwana N, et al (2011) Meningococcal disease in children in Merseyside, England: a 31 year descriptive study. PLoS One 6: e25957. 22016791 10.1371/journal.pone.0025957 PMC3189236

[17] Weiss DP, Coplan P, Guess H (2001) Epidemiology of bacterial meningitis among children in Brazil, 1997–1998. Rev Saude Publica 35: 249–255. 11486147 10.1590/s0034-89102001000300006

[18] Nascimento-Carvalho CM, Moreno-Carvalho OA (2004) Etiology of bacterial meningitis among children aged 2–59 months in Salvador, Northeast Brazil, before and after routine use of *Haemophilus influenzae* type B vaccine. Arq Neuropsiquiatr 62: 250–252. 15235726 10.1590/s0004-282x2004000200011

[19] Cordeiro SM, Neves AB, Ribeiro CT, Petersen ML, Gouveia EL, et al (2007) Hospital-based surveillance of meningococcal meningitis in Salvador, Brazil. Trans R Soc Trop Med Hyg 101: 1147–1153. 17681359 10.1016/j.trstmh.2007.06.012 PMC2042916

[20] Santos ML, Ruffino-Netto A (2005) [Meningococcal disease: epidemiological profile in the Municipality of Manaus, Amazonas, Brazil, 1998/2002]. Cad Saude Publica 21: 823–829. 15868040 10.1590/s0102-311x2005000300016

[21] Nascimento KA, Miranzi Sde S, Scatena LM (2012) Epidemiological profile of meningococcal disease in the State of Minas Gerais and in the Central, North, and Triangulo Mineiro regions, Brazil, during 2000–2009. Rev Soc Bras Med Trop 45: 334–339. 22760132 10.1590/s0037-86822012000300011

[22] Miranzi Sde S, de Moraes SA, de Freitas IC (2007) Impact of the *Haemophilus influenzae* type b vaccination program on HIB meningitis in Brazil. Cad Saude Publica 23: 1689–1695. 17572819 10.1590/s0102-311x2007000700021

[23] Zanella RC, Bokermann S, Andrade AL, Flannery B, Brandileone MC (2011) Changes in serotype distribution of *Haemophilus influenzae* meningitis isolates identified through laboratory-based surveillance following routine childhood vaccination against *H. influenzae* type b in Brazil. Vaccine 29: 8937–8942. 21945960 10.1016/j.vaccine.2011.09.053

[24] Braikat M, Barkia A, El Mdaghri N, Rainey JJ, Cohen AL, et al (2012) Vaccination with *Haemophilus influenzae* type b conjugate vaccine reduces bacterial meningitis in Morocco. Vaccine 30: 2594–2599. 22306854 10.1016/j.vaccine.2012.01.041

[25] Ribeiro GS, Lima JB, Reis JN, Gouveia EL, Cordeiro SM, et al (2007) Haemophilus influenzae meningitis 5 years after introduction of the *Haemophilus influenzae* type b conjugate vaccine in Brazil. Vaccine 25: 4420–4428. 17449150 10.1016/j.vaccine.2007.03.024

[26] Toscano CMO, Carmo EH (2009) Morbidade e mortalidade por doenças transmissíveis no Brasil. In: Brasil. Ministério da Saúde, editor. Saúde Brasil 2009: Uma análise da situação de saúde. Brasilia.

[27] McIntyre PB, O'Brien KL, Greenwood B, van de Beek D (2012) Effect of vaccines on bacterial meningitis worldwide. Lancet 380: 1703–1711. 23141619 10.1016/S0140-6736(12)61187-8

[28] Miller E, Andrews NJ, Waight PA, Slack MP, George RC (2011) Herd immunity and serotype replacement 4 years after seven-valent pneumococcal conjugate vaccination in England and Wales: an observational cohort study. Lancet Infect Dis 11: 760–768. 21621466 10.1016/S1473-3099(11)70090-1

[29] Hsu HE, Shutt KA, Moore MR, Beall BW, Bennett NM, et al (2009) Effect of pneumococcal conjugate vaccine on pneumococcal meningitis. N Engl J Med 360: 244–256. 19144940 10.1056/NEJMoa0800836 PMC4663990

[30] Brasil. Ministério da Saúde (2001) Manual de Normas de vacinação. Brasilia. 58 p.

[31] Brasil. Ministério da Saúde (2006) Manual dos Centros de referencia para imunobiologicos especiais. Brasilia. 190 p.

[32] Updated recommendations for use of meningococcal conjugate vaccines — Advisory Committee on Immunization Practices (ACIP), 2010. MMWR Morb Mortal Wkly Rep 60: 72–76. 21270745

[33] World Health Organization (2007) Pneumococcal conjugate vaccine for childhood immunization – WHO position paper. Weekly epidemiological record 82: 93–104. 17380597

[34] Brasil. Ministério da Saúde (2010) Introdução da vacina meningocóccica C (conjugada) no calendário de vacinação da criança: incorporação 2o semestre de 2010. assessed at: http://portal.saude.gov.br/portal/arquivos/pdf/it_meningo_implantacao.pdf

[35] Brasil. Ministério da Saúde (2010) Proposta para introdução da vacina pneumocócica 10-valente (conjugada) no calendário básico de vacinação da criança: incorporação março 2010. assessed at: http://portal.saude.gov.br/portal/arquivos/pdf/intro_pneumococica10_val_04_02_10_ver_final.pdf.

[36] Afonso ETM, Bierrenbach AL, Escalante JJC, Alencar AP, Domingues C, et al (2013) Impact of 10-valent pneumococcal vaccine on pneumonia hospitalization shortly after vaccine introduction in five Brazilian capital cities. Emerging Infectious Diseases (Print) epub ahead of print. 10.3201/eid1904.121198 PMC3647414 23628462

[37] Trotter CL, Ramsay ME, Gray S, Fox A, Kaczmarski E (2006) No evidence for capsule replacement following mass immunisation with meningococcal serogroup C conjugate vaccines in England and Wales. Lancet Infect Dis 6: 616–617; author reply 617–618. 17008169 10.1016/S1473-3099(06)70584-9

[38] Tanno LK, Ganem F, Demoly P, Toscano CM, Bierrenbach AL (2012) Undernotification of anaphylaxis deaths in Brazil due to difficult coding under the ICD-10. Allergy 67: 783–789. 22519410 10.1111/j.1398-9995.2012.02829.x

